# Liquid Phase Separation in High-Entropy Alloys—A Review

**DOI:** 10.3390/e20110890

**Published:** 2018-11-20

**Authors:** Nicholas Derimow, Reza Abbaschian

**Affiliations:** Department of Materials Science and Engineering, University of California, Riverside, CA 92521, USA

**Keywords:** high-entropy alloys, liquid phase separation, immiscible alloys, HEAs, multicomponent alloys, miscibility gaps, multi-principal element alloys, MPEAs, complex concentrated alloys, CCAs

## Abstract

It has been 14 years since the discovery of the high-entropy alloys (HEAs), an idea of alloying which has reinvigorated materials scientists to explore unconventional alloy compositions and multicomponent alloy systems. Many authors have referred to these alloys as multi-principal element alloys (MPEAs) or complex concentrated alloys (CCAs) in order to place less restrictions on what constitutes an HEA. Regardless of classification, the research is rooted in the exploration of structure-properties and processing relations in these multicomponent alloys with the aim to surpass the physical properties of conventional materials. More recent studies show that some of these alloys undergo liquid phase separation, a phenomenon largely dictated by low entropy of mixing and positive mixing enthalpy. Studies posit that positive mixing enthalpy of the binary and ternary components contribute substantially to the formation of liquid miscibility gaps. The objective of this review is to bring forth and summarize the findings of the experiments which detail liquid phase separation (LPS) in HEAs, MPEAs, and CCAs and to draw parallels between HEAs and the conventional alloy systems which undergo liquid-liquid separation. Positive mixing enthalpy if not compensated by the entropy of mixing will lead to liquid phase separation. It appears that Co, Ni, and Ti promote miscibility in HEAs/CCAs/MPEAs while Cr, V, and Nb will raise the miscibility gap temperature and increase LPS. Moreover, addition of appropriate amounts of Ni to CoCrCu eliminates immiscibility, such as in cases of dendritically solidifying CoCrCuNi, CoCrCuFeNi, and CoCrCuMnNi.

## 1. Introduction

### 1.1. Liquid Phase Separation

Liquid phase separation (LPS), a widely-observed phenomenon in metals, is related directly to the Gibbs free energy of the system, and the most prevailing cases are often two distinct immiscible liquids of varying compositions. Although there is often some degree of solubility between the alloying elements in a metallic system exhibiting LPS, each liquid will have its own equilibrium vapor pressure, such that the vapor pressures of both phases are the same, with a positive deviation from Raoult’s law. When positive deviations from Raoult’s law are large, phase segregation tends to occur.

The occurrence of liquid phase separation in an alloy can lead to heterogeneous microstructures, which may or may not be desirable depending on the intended application. For example, an alloy exhibiting liquid phase separation would not be suited for use as a structural material due to the heterogeneity of the microstructure; however, it may have potential use as a self-lubricating bearing material, such as the case with Cu-Pb. There have been several comprehensive reviews of immiscible metal systems of common alloys about the phenomenon [1,2,3,4,5]. Therefore, the scope of this review will focus particularly on the liquid phase separation in the high-entropy alloy (HEA), complex concentrated alloy (CCA), and multi-principal element alloy (MPEA) systems.

### 1.2. Thermodynamics of Liquid Phase Separation

Factors such as positive deviations from Raoult’s law, positive heat of mixing, and atomic size mismatch in some cases do not overcome the entropy term in the overall Gibbs free energy and cause overall immiscibility in the liquid, as is the case of miscibility between Au-Bi [5]. B. Mott in the late 1950s put together a review of the immiscible liquid metal systems, as well as the corresponding thermodynamic data for each material at the time. The immiscible alloys Mott compiled in the study contained many of the known immiscible binary monotectic alloys of the time [1]. Nearly ten years later, Mott compiled another review detailing the thermodynamics of these metal systems, as well as provided models for predictions of immiscibility in metals [2].

In Table 1, we provide a non-exhaustive table of binary alloys with miscibility gaps in the liquid state. The table expands the tables from Mott’s reviews [1,2] by adding immiscible alloys from binary phase diagrams provided by the Centre for Research in Computational Thermochemistry using the FactSage thermochemical software databases [6].

The molar Gibbs free energy of a system of stable unmixed liquids is represented additively via the atomic fraction of the free energies of the constituent liquids,

(1) $$\begin{array}{c} G_{A\;+\;B\;+\;C\;\ldots }^{L}=\sum_{i=A,B,C\ldots }x_{i}G_{i}^{L} \end{array}$$

where $x_{i\;=\;A,B,C\ldots }$ are the molar fractions of elements A, B, C, etc. The molar Gibbs free energy of mixing is classically defined as,

(2) $$\begin{array}{c} \Delta G_{\mathrm{mix}}=\Delta H_{\mathrm{mix}}-T\Delta S_{\mathrm{mix}} \end{array}$$

where the entropy of mixing is given as,

(3) $$\begin{array}{c} \Delta S_{\mathrm{mix}}=-R\sum_{i}x_{i}\ln x_{i} \end{array}$$

and the enthalpy of mixing is:

(4) $$\Delta H_{\mathrm{mix}}=\sum_{i=1,i\neq j}^{n}\Delta H_{x_{i},x_{j}}^{\mathrm{mix}}$$

where $\Delta H_{x_{i},x_{j}}^{\mathrm{mix}}$ is the interatomic interaction between concentrations of “*i* ” and “*j*” elements in the system. Immiscible alloys typically have a positive value of $\Delta H_{\mathrm{mix}}$, which implies a preference of nearest neighbors of similar atoms as opposed to compound formation with different atoms. Many of the immiscible binary systems can be categorized by their liquid state miscibility gaps and positive enthalpy of mixing, $\Delta H_{\mathrm{mix}}$, of which extensive thermodynamic treatments are presented in [3,5].

The region of a phase diagram where there is non-mixing of the constituents is defined as a miscibility gap. The liquid miscibility gap in many of the monotectic binary systems assumes a dome-like shape; the shape and location of which may shift with the addition of more alloying elements. For example, one of the most well-studied ternary systems with a stable liquid miscibility gap is the Co-Cu-Fe system [7,8,9,10,11,12,13,14,15,16,17], while with equiatomic additions of Cr and Ni, the CoCrCuFeNi high-entropy alloy solidifies dendritically from a single-phase liquid, as observed by Yeh et al. in 2004 [18].

**Figure 1:**
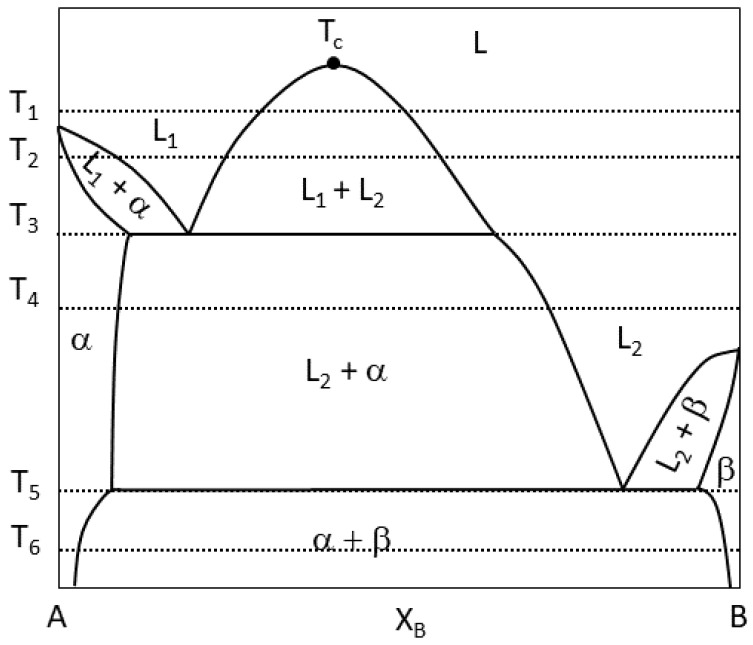
Generalized equilibrium monotectic binary phase diagram.

A generalized equilibrium monotectic phase diagram is presented in Figure 1, where the miscibility gap in the liquid state is present as a dome with label L${}_{1}$ + L${}_{2}$. The size and width of the immiscibility gap varies from system to system; however, the concept is the same. That is, cooling the alloy system from a liquid state in the concentrations that fall within the miscibility gap will lead to the liquid decomposing into two compositionally different liquids, the temperature of which is known as the critical temperature (labeled T${}_{c}$ in Figure 1).

**Figure 2:**
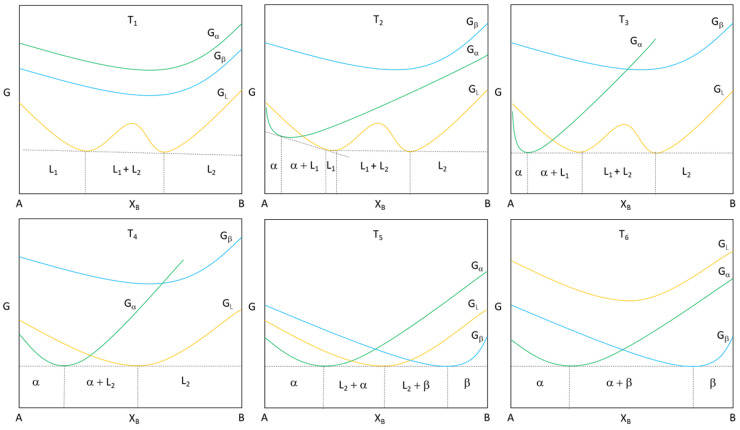
Gibbs free energy corresponding to the monotectic phase diagram.

As the temperature decreases to T${}_{1}$ in Figure 1, the entropy term TΔS is smaller than the enthalpy of mixing $\Delta H_{\mathrm{mix}}$ in the free energy of the system (Figure 2); therefore, the free energy of the liquid G${}_{L}$ with respect to concentration of the B element in A will also assume a dome shape, presented in Figure 1. If the temperature T${}_{1}$ is held, the equilibrium phases will be L${}_{1}$, L${}_{1}$ + L${}_{2}$, or L${}_{2}$ dependent on composition X${}_{B}$. Cooling the system through T${}_{2}$ until the monotectic temperature, T${}_{3}$, the monotectic reaction will take place, and we will start to see α precipitate out of the liquid as L${}_{1}$ is no longer stable until we reach T${}_{4}$, where the remaining equilibrium phases are the solidified α and liquid L${}_{2}$. Cooling through the eutectic temperature at T${}_{5}$ to reach T${}_{6}$, we are ultimately left with (α+β) solid phases.

Due to the lack of experimental data for mixing enthalpies of many binary alloys, a model for generating approximate mixing enthalpies was first developed by Miedema et al. in 1973 [19], which uses the electron density at the Wigner–Seitz cell boundary and the chemical potential of electronic charge of pure metals as input and can be written as $\Delta H_{\mathrm{mix}}=\sum_{i=1,i\neq j}^{n}\Delta H_{c_{i},c_{j}}^{\mathrm{mix}}$. This model was used by Takeuchi et al. in 2005 for the classification of bulk metallic glasses by atomic size difference and heat of mixing [20] and later revisited by Takeuchi in 2010 [21] for mixing enthalpies of binary alloys, which includes an additional model for sub-regular solutions [22]. The $\Delta H_{\mathrm{mix}}$ of the binary alloys from [21] serve as a starting point for many of the recent calculations of $\Delta H_{\mathrm{mix}}$ for HEAs, MPEAs, and CCAs. Using the calculated binary mixing enthalpies ($\Delta H_{\mathrm{mix}}$) from Takeuchi et al. [21], $\Delta H_{\mathrm{mix}}$, much of the values used for determining the mixing enthalpies for HEAs/CCAs/MPEAs were calculated using Equation (4) where $\Delta H_{x_{i},x_{j}}^{\mathrm{mix}}=4\Omega_{0_{ij}}x_{i}x_{j}$ for the $i^{\mathrm{th}}$ and $j^{\mathrm{th}}$ elements at A${}_{0.50}$B${}_{0.50}$ concentrations from the tables in [21]. The values for $c_{i}$ and $c_{j}$ are the normalized atomic concentrations in the multicomponent alloy.

### 1.3. Metastable Liquid Phase Separation

**Figure 3:**
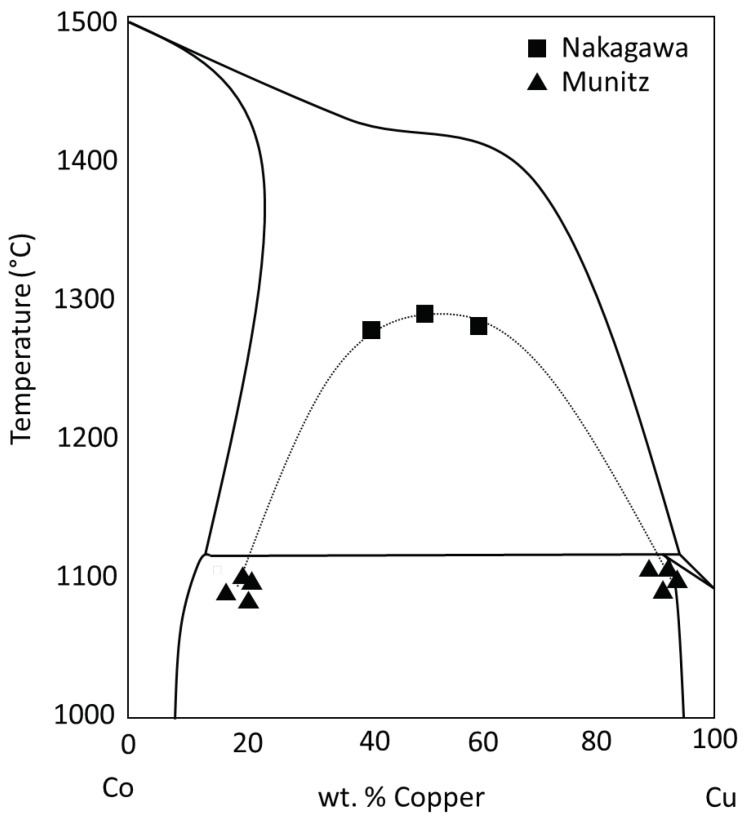
The Co-Cu phase diagram with the dashed line indicating the metastable liquid miscibility gap beneath the liquidus curves.

Unlike the stable liquid state immiscibility observed in the monotectic binary alloys, there are certain cases where a completely miscible liquid alloy can de-mix in the presence of impurities or when supercooled below the freezing temperature of the alloy, as demonstrated for Co-Cu and Cu-Fe by Nakagawa in 1958 [23]. Since then, there has been an enormous amount of LPS studies on metastable Co-Cu [8,15,16,24,25,26,27,28,29,30,31,32,33,34] and Cu-Fe [15,35,36,37,38,39,40,41,42,43], as well as the stable LPS that occurs in the combination of all three elements in Co-Cu-Fe [7,8,9,10,11,12,13,14,15,16,17]. Metastable liquid phase separation is defined as the liquid phase separation that occurs when undercooling an alloy such that it enters a miscibility gap that would not have been observed if solidified via conventional methods, presented in the phase diagram from [25] in Figure 3. These studies have shown that when undercooling past freezing, the single-phase alloy liquid will then split into two liquids (L1 + L2), specifically in these cases, into Cu-rich and Cu-lean liquids, which solidify often as spherical globules trapped in the frozen regions of the other liquid (the microstructure of such will be discussed later). This metastable LPS implies that there exists a dome shape similar to the monotectic alloys beneath the liquidus curves in their respective equilibrium phase diagrams.

### 1.4. High-Entropy Alloys

The discovery of the high-entropy alloys (HEAs) [18,44,45,46,47,48] has inspired an enormous amount of research into multicomponent alloy design. Since their inception, there have been numerous reviews [49,50,51,52,53,54,55,56,57,58,59,60] and several books [61,62,63,64,65,66] that summarize the state-of-the-art for the materials community. These reviews compile and assess the microstructural developments, mechanical properties, crystallography, and thermodynamics of the high-entropy, complex concentrated, and multi-principal element alloy systems.

Many of the ternary alloys that exhibit liquid phase separation contain Cu, as it has a low affinity for mixing with other elements; however, there are a number of other non-Cu-containing ternary alloys with liquid miscibility gaps as well. Table 2 is a non-exhaustive list of the studied ternary alloys with liquid phase miscibility gaps, many of which contain Cu. As was the case with many binary alloys, the $\Delta H_{\mathrm{mix}}$ of these systems are typically positive. One can think of the HEAs/CCAs/MPEAs as the addition of alloying elements to preexisting ternary alloys, some of which may actually contain a stable miscibility gap in the liquid, which is the case with CoCrCu [67] and many of the HEAs that contain Co, Cr, and Cu in equal parts with respect to the other alloying elements in the HEA. It would appear that the increase in the entropy of mixing $\Delta S_{\mathrm{mix}}$ with additional alloying elements stabilizes the solution; however, this strongly depends on the enthalpy of mixing $\Delta H_{\mathrm{mix}}$, as well as other factors that determine miscibility [58].

One of the first HEAs contained equal parts Co, Cr, Fe, Mn, and Ni, often written alphabetically as CoCrFeMnNi and referred to as the “Cantor alloy” after the alloy’s inventor Brian Cantor [44]. Subsequent studies of the Cantor alloy involved substitution of various elements in place of the original five equiatomic elements in the alloy. One of the most popular substitutions is often Cu for Mn [18], as well as the addition of Al to create the widely-studied AlCoCrCuFeNi HEAs [85,86,87]. Since their inception, the majority of the high entropy alloys that are synthesized consist mostly of the 3D transition metals with other elements substituted intothe well-studied systems in the search for new stable phases, mechanical properties, and reproducible microstructures [58]. There have been several research papers that prescribe methodologies for computationally screening HEAs for those that are likely to create single-phase solid solutions [88,89,90,91,92,93,94,95,96]. A useful criteria for HEA/CCA/MPEA research is to know whether or not the combination of elements in a proposed HEA system will even mix in the liquid phase, as LPS traditionally is an unwanted phenomena when designing new materials with the goal of enhancing mechanical properties. If a single-phase liquid can be obtained from the proposed combination of elements, then the solidification microstructures will result in more uniformity.

There is much debate as to what exactly constitutes a high-entropy alloy (HEA), complex concentrated alloy (CCA), or multi-principal element alloy (MPEA). In essence, the core idea is very similar behind each definition: multicomponent, “baseless” alloys greater than three elements designed with the goal of surpassing the mechanical properties of traditional alloys. Whether or not which composition will solidify into a single, duplex, or multiple phases is not what this review is concerned with, but rather the observance of liquid phase separation in these multicomponent alloy systems.

Approximately 85% of HEAs in the literature to date are made up of predominantly 3D transition metals, many of which contain Al [58]. As it is impossible to visualize the phase diagram space of multicomponent alloy systems that contain 5+ elements, it can be difficult to know whether these alloy systems will phase separate in the liquid. Many of the well-studied HEA systems, such as CoCrCuFeNi for example [45], contain equiatomic CoCrCu, which has a very large liquid miscibility gap [67,70].

## 2. Solidification Microstructures

### 2.1. Dendritic Microstructure

Alloy solidification morphology and as-cast microstructure can have many forms depending on the solidification process. The most common microstructures of a solidified alloy can vary from plane front solidification, dendritic morphology, and eutectic microstructures, among others. Many of the solidification microstructures present in HEA literature consist of dendritic growth, which is typically indicative of crystal growth from a liquid with an imposed thermal gradient, as is the case with most arc-melting processes. Dendrites are solid tree-like, branching cellular structures that grow from a liquid phase. The conglomeration of atoms during solidification typically forms a nucleus of spherical shape, which then becomes unstable due to perturbations. The solid shape then begins to express the preferred growth directions of the underlying crystal and consumes atoms from the overall liquid to form a stable solidifying phase [97]. The liquid that is leftover after dendritic solidification is referred to as the interdendritic liquid, which solidifies last, and is referred to as the interdendritic region or interdendrite. The preferred dendritic growth directions for most cubic systems (FCC/BCC) are in the <100> directions, which leads the secondary dendrite arms to grow perpendicular from the primary arm. This is often an easy way to differentiate between the cubic and noncubic crystal structure of the dendritic phase.

**Figure 4:**
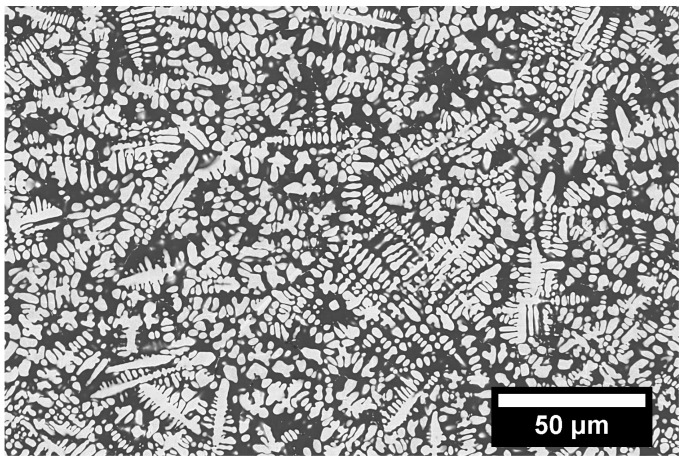
Backscattered electron image of an as-cast AlMoNi alloy with a dendritic microstructure.

The typical dendritic microstructure of an electromagnetically-levitated and solidified alloy is presented in Figure 4. The dendritic morphology usually indicates that the microstructure evolved from a single-phase liquid if the dendritic morphology is uniform; however, if the alloy has a small volume fraction of LPS, the remaining L2 globules may be pushed to the edge of the sample by the growing dendrites. There have been cases where liquid phase separation has occurred in the interdendritic liquid after the growth of primary dendrites [98,99]; however, the general morphology of dendritic microstructures indicates that there was no large-scale liquid-liquid immiscibility between the alloying elements.

Many HEAs/CCAs/MPEAs solidify with a duplex microstructure, where the dendritic and interdendritic regions have large compositional and crystallographic differences [53,58,59,60,64]. These have been shown to have interesting mechanical properties; however, they have yet to surpass the mechanical properties of commercial alloys.

### 2.2. Microstructures Resulting from Liquid Phase Separation

**Figure 5:**
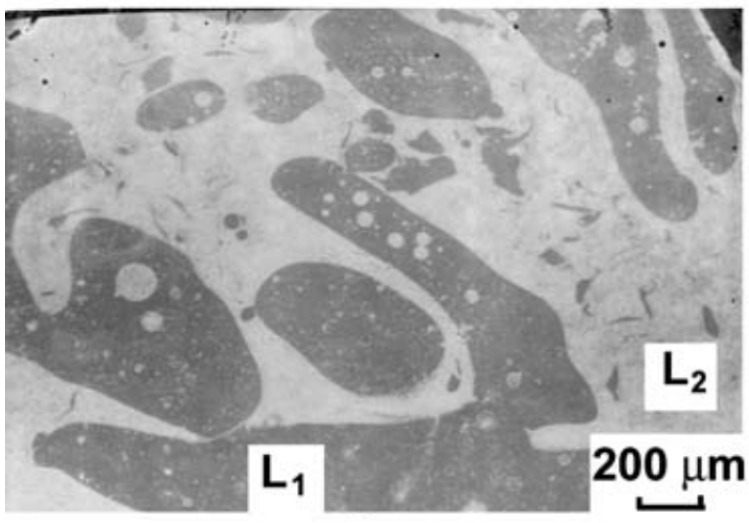
SEM image of liquid phase separation and emulsion of two immiscible liquids L1 and L2 in an undercooled CoCuFe alloy. Image presented with permission from the authors in [16].

**Figure 6:**
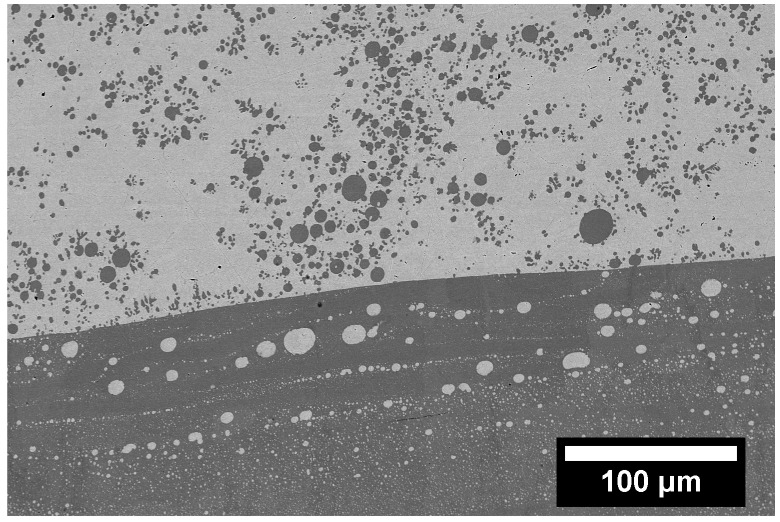
Backscattered electron image displaying emulsion of CoCr-rich (darker) and Cu-rich (lighter) liquids in an as-cast alloy of CoCrCu.

**Figure 7:**
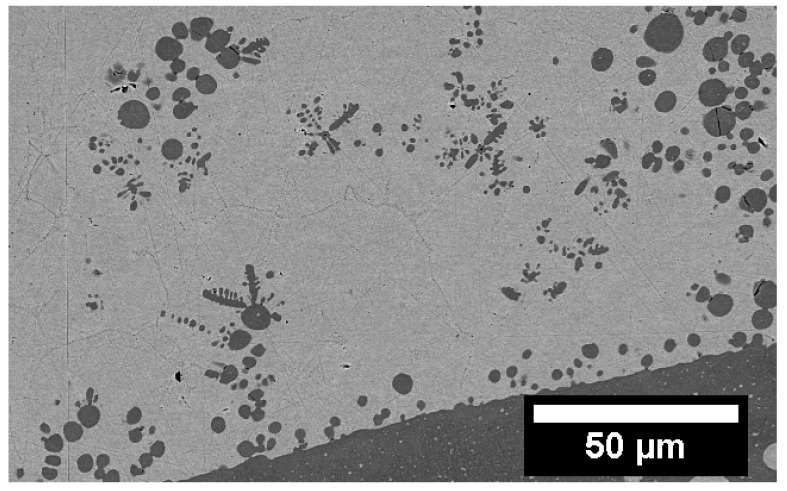
Backscattered electron image displaying emulsion and protrusions of the CoCr-rich (darker) phase into the Cu-rich (lighter) phase in an as-cast alloy of CoCrCu.

Alloys that undergo either stable or metastable LPS also have very distinct microstructures that can vary based on the solidification process. Slow cooling rates paired with a static environment can lead to the liquids separating. If the system is a little more dynamic, such as in the case of casting, the process can lead to trapping of the primary liquids in one another, referred to as emulsion (Figure 5). The separated liquids tend to be trapped as spherical globules inside the other liquid, and solidify as such, as is the case of the equiatomic CoCrCu alloy presented in Figure 6. As these liquids can be slightly different in composition than the primary liquid, they are referred to as secondary liquids. Based on morphology alone, one can distinguish the first phase to solidify from the interface between the the two liquids, as the higher melting point liquid will solidify at a higher temperature and will most of the time solidify with protrusions into the other liquid, as is the case with the solidification of CoCrCu presented in Figure 7. The backscattered electron images in Figure 6 and Figure 7 display emulsion of the lighter (Cu-rich) and darker (CoCr-rich) liquids, as well as small protrusions coming from the CoCr-rich secondary liquid in Figure 7.

There have been several efforts to create uniform microstructures of immiscible liquid melts, such that the LPS is evenly distributed. These techniques include free directional and directional solidification [100], rheomixing [101], microgravity experiments [28,102,103], electromagnetic levitation processing [40], and rapid solidification [104]. Much of this work is aimed at the tailoring of the immiscible liquid droplets such that the microstructure has a uniform spread of the immiscible phase [3]. There have also been experiments aimed at the suppression of LPS with additional alloying elements [105].

**Figure 8:**
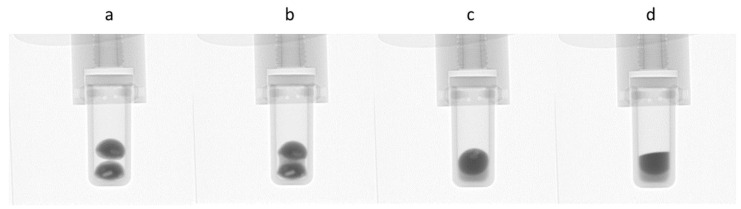
Melting and liquid phase separation of stacked CoCrCu samples. (**a**) During initial heating, the two as-cast buttons are intact; (**b**) the Cu-rich phase melts first between 1075 and 1100 °C and (**c**) pools at the bottom of the crucible; (**d**) the Cu-lean phase fully melts upon heating to 1500 °C and stacks based on density due to the influence of gravity. Images displayed with permission from the authors in [72].

**Figure 9:**
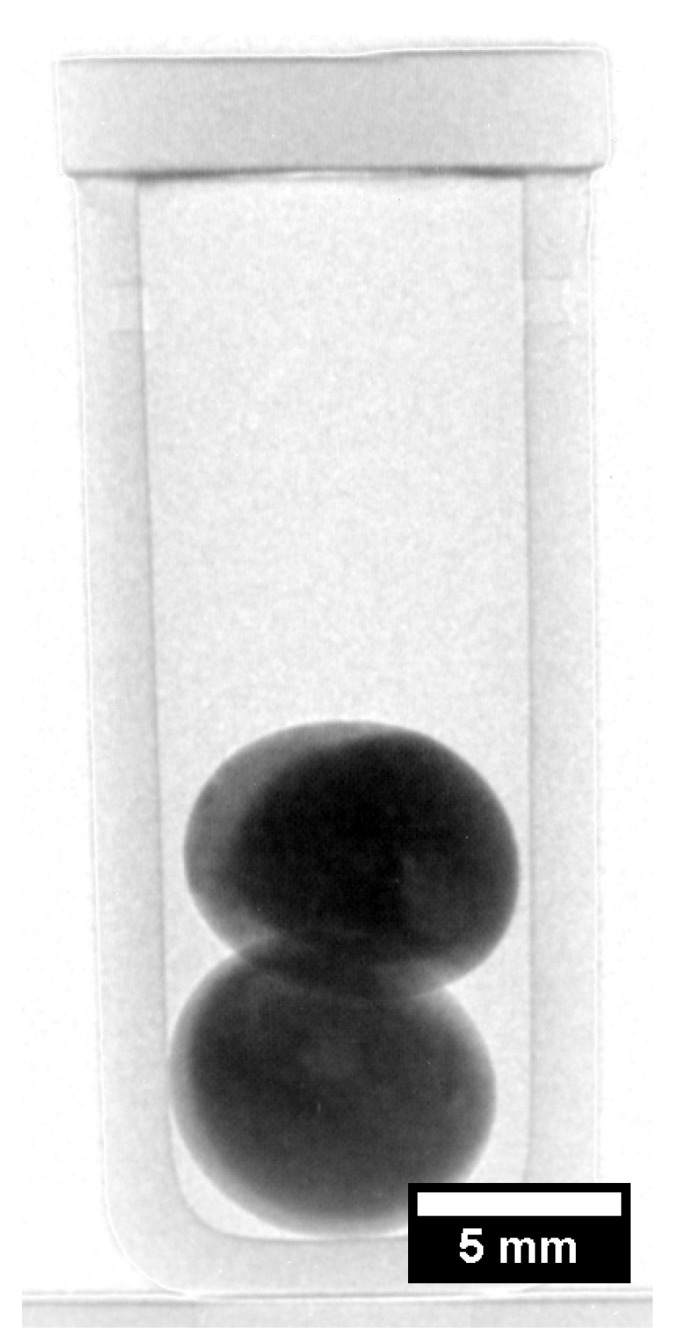
Neutron radiograph of two stacked arc-melted CoCrCu buttons in an alumina crucible with brighter regions corresponding to Cu-rich phases and darker regions corresponding to CoCr-rich phases. Image displayed with permission from the authors in [72].

Traditionally, observing LPS in metallic systems was done via post-mortem analysis via metallography and microscopy, as metals are not transparent to light and have a very small transparency for X-rays. Recently, through the use of neutron transmission imaging techniques, the direct observation of liquid phase separation in metals was made possible via neutron radiographs taken during heating and cooling of immiscible CoCrCu alloys [72]. These experiments show for the first time an in situ observation of macroscopic LPS in metals and can be applied to any metallic system such that the neutron transmission through the each phase can provide enough contrast between them. Figure 8 displays neutron radiographs of two stacked CoCrCu arc-melted buttons in a small Al${}_{2}$O${}_{3}$ crucible with an inner diameter of 8 mm. Prior to the in situ testing, the arc-melted CoCrCu buttons underwent stable LPS and solidified with very heterogeneous Cu-rich and Cu-depleted regions, as presented in Figure 9. The resulting as-cast button consists of a non-uniform mix of the two solidified Cu-rich and Cu-depleted phases and can be seen as the lighter (Cu-rich) and darker (Cu-depleted) regions in Figure 8a. During melting (Figure 8b–d), the two liquid phases separate and stack according to density (Cu being the lighter contrast, more dense liquid phase). Note that the solid Cu-rich and CoCr-rich phases were already separated in the arc-melted buttons, but at a finer scale. The stable liquid phases then agglomerated and separated at the macro-scale. A full sequence of images in the form of a movie can be found in [72].

## 3. High-Entropy Alloys Exhibiting Liquid Phase Separation

### 3.1. HEAs Containing Cu

The first occurrence of LPS in HEAs was observed by Hsu et al. in 2007 with a study of the alloying behavior of AlCoCrCuNi-based HEAs with additions of Fe, Ag, and Au [106]. The addition of Ag to the AlCoCrCuNi HEA to create AgAlCoCrCuNi was found to phase separate in the liquid, which resulted in the solidification microstructure consisting of Cu-rich globules embedded in Cu-depleted phases, contrary to the typical dendritic solidification microstructures observed for AlCoCrCuNi. Hsu suggested that in order to achieve effective mixing in the liquid, the $\Delta H_{\mathrm{mix}}$ for atom pairs should not exceed 10 kJ/mol and that “mutual interaction between elements, based on their mixing enthalpies, should be taken into account when designing high-entropy alloys” [106].

These alloys were then revisited by Munitz et al. in 2013 where AgAlCoCrCuNi and AgAlCoCrCuFeNi were synthesized to study the melt separation behavior [107]. It was observed that the Cu-rich immiscible liquid tended to flow to the bottom of the buttons during arc-melting, as well as residual Cu-rich liquid being trapped in the interdendritic region of the Cu-depleted dendritic phase. Undercooling experiments were also carried out for a similar alloy of Al${}_{1.8}$CoCrCu${}_{3.5}$FeNi; however, no metastable liquid miscibility gap was found at the undercoolings obtained in the study (∼150 K) [107].

A similar alloy composition of AlCoCrCuFeNiSi${}_{0.5}$ doped with Y${}_{2}$O${}_{3}$ was synthesized via laser cladding with the intention to form a core-shell structure in HEAs, inspired by the LPS observed in HEAs and binary monotectics. The undoped AlCoCrCuFeNiSi${}_{0.5}$ did not undergo LPS, while the addition of 1 wt.% nanosized Y${}_{2}$O${}_{3}$ caused the liquids to separate into egg-like globules of Cu-rich liquids inside the Cu-depleted liquid [108].

Recent studies into Co-free Al${}_{2.2}$CrCuFeNi${}_{2}$ revealed what the authors referred to as anomalous “sunflower-like” solidification microstructures [109], where it was suggested that LPS occurs in the no longer stable-depleted interdendritic liquid, occurring due to changes in the composition. Munitz et al. suggested that the liquid phase separation is due to constitutional changes and not temperature changes, where the authors referred to this phenomena as “constitutional LPS” (CLPS) [99]. For the Al${}_{2.2}$CrCuFeNi${}_{2}$ alloy, constitutional LPS occurred in the interdendritic liquid, where the interdendritic liquid decomposed into a CrFe-rich L${}_{1}$ and a Cu-rich L${}_{2}$. As the dendritic skeleton was already formed, the heavier Cu-rich liquid accumulated in the interdendritic region and the cast bottom, while the CrFe-rich spheres underwent solidification emulsified in the Cu-rich liquid.

**Figure 10:**
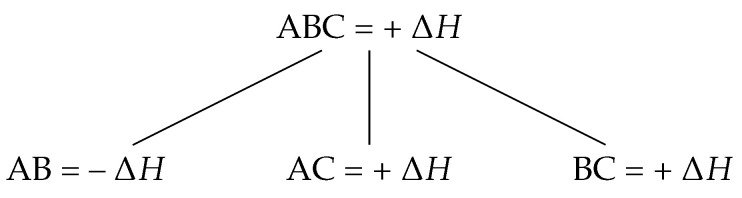
Tree diagram representing the probability of clustering based on the mixing enthalpies of binary combinations of elements ABC.

A large study of several HEAs by Munitz et al. in 2017 was undertaken to explore the effects of Al, Co, Cr, Ni, Ti, and V on the miscibility gap temperature of several HEA systems. It was shown that Al, Co, Ni, and Ti lowered the miscibility gap temperature, while Cr, V, and Nb raised the miscibility gap temperature and increased LPS in these systems, the alloys of which are found in Table 3. Many of the HEAs studied by Munitz et al. contained equiatomic CoCrCu, which was experimentally determined to have a large liquid miscibility gap [67]. It is peculiar that systems such as CoCrCuFeNi will solidify dendritically [18], while similar alloys of CoCrCu [67,71,72] and CoCuFe [7,8,9,10,11,12,14,15,16,17,73] have been shown to have large liquid miscibility gaps. A recent study by Derimow et al. investigated the solidification microstructures of equiatomic CoCrCu with added Fe, Mn, Ni, V, FeMn, FeNi, FeV, MnNi, MnV, and NiV to the composition. It was found that only three of the alloys solidified dendritically (CoCrCuNi, CoCrCuFeNi, and CoCrCuMnNi), while the remaining combinations underwent stable LPS [71]. Derimow et al. also suggested that the positive mixing enthalpy of each of the systems was responsible for the LPS and presented a tree diagram for approximating the likelihood of which elements will cluster together in the melt, presented in Figure 10.

The tree diagram in Figure 10 indicates that AB atoms are more likely to cluster than ABC, AC, or BC, thereby rejecting the C element. This can be seen in case of the ternary CoCrCu, where the LPS consists of CoCr-rich and Cu-rich liquids [67,71] and solidifies with the microstructure shown in Figure 6.

Table 3 compiles the multicomponent alloys found in the literature that have been shown to undergo LPS. Each of these systems have a solidification morphology similar to the microstructure presented in Figure 6. From this table, the common component in all but one of these systems is equiatomic Cu. This is in part due to the positive mixing enthalpy Cu has with many of the alloying elements in the system. It should be noted that although Ni appears to promote miscibility in Cu-containing HEAs, this may be ineffective if the repulsion between Cu and the majority of the alloying elements is too great.

### 3.2. CoCrCuFeNi

One of the seminal HEAs synthesized by Yeh et al. was the CoCrCuFeNi HEA [18]. Along with the Cantor alloy (CoCrFeMnNi) [44], these alloys served as many starting points for the addition and subtraction of alloying elements. The first study to note the liquid phase separation in HEAs in a similar composition of AgAlCoCrCuNi attributed the presence of LPS to the positive mixing enthalpies between Ag and the rest of the alloying elements [106].

An induction melting study by Wu et al. involving CoCrCuFeNi demonstrated the first occurrences of LPS in this alloy [116]. In Wu’s study, several combinations of the CoCrCuFeNi alloy with varied Fe and Ni were studied to investigate the effects on the microstructure and crystallography of the system. When Fe and Ni were varied to create CoCrCuFe${}_{0.5}$Ni and CoCrCuFeNi${}_{0.5}$, spherical Cu-rich separations were observed by post-mortem analysis of the solidified samples [116]. The authors rationalized that the two alloy compositions with positive mixing enthalpies were too great to be overcome by the entropy of the system, which would thereby lower the overall Gibbs energy of the system in the molten state.

Previous studies of supercooling and rapid solidification via electromagnetic levitation melting by Elder et al. characterized large undercoolings of 150 K for Cu-Fe and 75 K for Co-Cu that produce the LPS microstructures for these metastable alloys [120]. Elder et al. listed several techniques to achieve high undercoolings such as melt emulsification, melting in molten slag or fused silica (glass fluxing), free fall in a drop tube, and electromagnetic levitation techniques to achieve undercooled temperatures [120]. Using the molten fused silica technique, rapid solidification studies of CoCrCuFeNi by Liu et al. were carried out to investigate rapid solidification effects on the microstructure and phase stability of CoCrCuFe${}_{x}$Ni HEAs (where x = 1, 1.5, and 2.0) [115]. It was found that LPS occurred in all three compositions below the critical undercooling temperature, $\Delta T_{\mathrm{crit}}$, where $\Delta T_{\mathrm{crit}}^{x=1.0}$ = 160 K, $\Delta T_{\mathrm{crit}}^{x=1.5}$ = 190 K, and $\Delta T_{\mathrm{crit}}^{x=2.0}$ = 293 K. When ΔT > $\Delta T_{\mathrm{crit}}$, the microstructure is consistent with that of LPS, such that there were Cu-rich spheres present throughout the material. Due to the dendritic solidification behavior of CoCrCuFeNi through regular solidification routes [18], this alloy is classified as having a metastable liquid phase miscibility gap that is present when undercooled past $\Delta T_{\mathrm{crit}}$ [115]. The metastable liquid miscibility gap was also confirmed by Wang et al. for CoCrCuFeNi, where the authors also achieved an exceptionally high degree of undercooling of 381 K (0.23T${}_{m}$) for the alloy [117]. The $\Delta T_{\mathrm{crit}}$ for equiatomic CoCrCuFeNi was also studied by Guo et al., and it was found that metastable LPS occurs when ΔT > $\Delta T_{\mathrm{crit}}$ = 100 K [118]. The authors also show that the yield strength and elongation of equiatomic CoCrCuFeNi significantly decrease when the alloy undergoes liquid phase separation due to the non-uniformity of the resultant microstructure [118].

Recently, Wang et al. showed that with the addition of 3 at.% Sn to CoCrCuFeNi, the alloy undergoes the same characteristic liquid phase separation when undercooled past $\Delta T_{\mathrm{crit}}$ = 100 K [121]. The study showed that the LPS produced an increase in hardness of the Cu-depleted phases due to the separation of Cu and Sn in the liquid [121].

Previous studies on similar alloys have shown that Mo improves the strength in the AlCoCrFeNi and AlCoCrCuFeNi HEAs [122,123,124]. However, it has been shown that with varied Cu concentrations in CoCrCu${}_{x}$FeMoNi where x ≥ 0.5, LPS occurs in a similar fashion to the other Cu-containing HEAs [114]. The addition of Mo to the CoCrCuFeNi alloy was investigated by Wu et al. to elucidate the solidification process in these alloys, as there had been a lack of thorough studies on the solidification microstructures of these alloys [114]. Due to the Cu-rich sphere emulsion in Cu-depleted phases, likely due to the positive mixing enthalpy between Cu and the remaining alloying elements, Wu et al. suggested that the $\Delta H_{\mathrm{mix}}$ criteria for the prediction of single phase formation be amended to include the possibility of liquid phase separation in the liquid when $\Delta H_{\mathrm{mix}}$ > 0. In order to further study the Mo-containing HEAs, Peng et al. synthesized a Co-depleted CrCu${}_{x}$FeMo${}_{y}$Ni HEA to further elucidate the effects of the large positive $\Delta H_{\mathrm{mix}}$ between Cu and Mo on the solidification process and microstructure. It was found that Cu-rich and Cu-depleted LPS occurs in the CrCu${}_{x}$FeMo${}_{y}$Ni when x and y = 0.5 and 1, attributed to $\Delta H_{\mathrm{mix}}$ > 0 for these alloy combinations [119].

## 4. Closing

The field of high-entropy alloys, complex concentrated alloys, and multi-principal element alloys continues to grow, with new studies producing valuable insights for the materials community with the overarching goal of creating new alloys that exceed the properties of conventional materials. This relatively new class of material is not much different from the conventional alloys, being that they are still subject to the same thermodynamic rules that are imposed on them. The main caveats are that with the increase of alloying elements, orthogonal element phase diagram visualization becomes impossible; therefore, creative ideas are warranted to help understand the nature of the solidification of these alloys. Positive mixing enthalpy, if not compensated by the entropy of mixing, will cause liquid phase separation. It appears that Co, Ni, and Ti promote miscibility in multicomponent alloys, while Cr, V, and Nb will raise the miscibility gap temperature and increase LPS. Moreover, for equiatomic CoCrCu, which has a large liquid miscibility gap, the addition of appropriate amounts of Ni eliminates immiscibility. The indication of such an example is the CoCrCuFeNi alloy, which will solidify dendritically, while similar alloys of CoCrCu and CoCuFe show strong immiscibility. Moreover, when Fe, Mn, Ni, V, FeMn, FeNi, FeV, MnNi, MnV, and NiV are added to to equiatomic CoCrCu, only three of the alloys solidify dendritically (CoCrCuNi, CoCrCuFeNi, and CoCrCuMnNi), while the remaining combinations undergo stable LPS. In the case of CoCrCuNiV, it appears that the addition of Ni in equiatomic amounts was not enough to overcome the positive mixing enthalpy interaction between Cu and V, as the CoCrCuV alloy also exhibits stable LPS. From the table of listed multicomponent alloys that undergo LPS, Cu is found in all but one of these combinations, which indicates that Cu containing HEAs may contain a metastable liquid miscibility gap such as the case with CoCrCuFeNi.

## Figures and Tables

**Table 1:** Binary systems that contain a stable miscibility gap in the liquid state.

Ag-B	Au-Ru	Bi-V	Ce-Cr	Cs-Fe	Fe-Na	K-Mo	Li-Tb	Na-Y	Sr-Tm
Ag-Co	B-Ge	Bi-Zn	Ce-Eu	Cu-K	Fe-Pb	K-Nd	Li-Ti	Na-Yb	Sr-V
Ag-Cr	B-Sn	C-Cu	Ce-K	Cu-Mo	Fe-Rb	K-Ni	Li-V	Na-Zn	Sr-Y
Ag-Fe	Ba-Ce	C-Sn	Ce-Li	Cu-Na	Fe-Sn	K-Pb	Li-Yb	Na-Zr	Sr-Zr
Ag-Ir	Ba-Cr	Ca-Cd	Ce-Mo	Cu-Pb	Fe-Sr	K-Pm	Li-Zr	Nd-Sr	Tb-Ti
Ag-K	Ba-Fe	Ca-Ce	Ce-Na	Cu-Ru	Fe-Tl	K-Pr	Lu-Na	Nd-Ti	Tb-V
Ag-Mn	Ba-Gd	Ca-Cr	Ce-Sr	Cu-Se	Ga-Hg	K-Sc	Lu-Sr	Nd-V	Te-Tl
Ag-Nb	Ba-K	Ca-Dy	Ce-Ti	Cu-Tl	Ga-Pb	K-Sm	Lu-V	Nd-Yb	Th-U
Ag-Nb	Ba-La	Ca-Er	Ce-U	Cu-Tu	Ga-Te	K-Sr	Lu-Yb	Ni-Pb	Ti-Yb
Ag-Ni	Ba-Mn	Ca-Fe	Ce-V	Cu-U	Ga-Tl	K-Tb	Mg-Mn	Ni-Sr	Tl-Zn
Ag-Os	Ba-Nd	Ca-Gd	Ce-Zr	Cu-V	Ge-Tl	K-Ti	Mg-Mo	Ni-Tl	Tm-V
Ag-Os	Ba-Pm	Ca-Ho	Co-In	Cu-W	Gd-K	K-Tm	Mg-Na	Pb-Se	V-Y
Ag-Rh	Ba-Pr	Ca-K	Co-K	Cr-Pb	Gd-Li	K-V	Mg-Nb	Pb-Si	V-Yb
Ag-Rh	Ba-Ru	Ca-La	Co-Li	Cr-Sn	Gd-Mo	K-Y	Mg-Ru	Pb-Zn	W-Zn
Ag-Se	Ba-Sc	Ca-Lu	Co-Pb	Dy-K	Gd-Na	K-Yb	Mg-Ru	Pb-Zr	
Ag-Ta	Ba-Sm	Ca-Mn	Co-Tl	Dy-Li	Gd-Sr	K-Zn	Mg-Ta	Pm-Sr	
Ag-U	Ba-Ti	Ca-Na	Cr-Dy	Dy-Na	Gd-Ti	K-Zr	Mg-Ti	Pm-Ti	
Ag-V	Ba-Y	Ca-Nd	Cr-Er	Dy-Sr	Gd-V	La-Li	Mg-W	Pm-V	
Ag-W	Ba-Zr	Ca-Pm	Cr-Eu	Dy-Ti	Gd-Yb	La-Mn	Mg-Zr	Pr-Sr	
Al-Bi	Be-K	Ca-Pr	Cr-Gd	Dy-V	Hg-Nb	La-Na	Mn-Na	Pr-Ti	
Al-Cd	Be-Li	Ca-Ru	Cr-K	Er-K	Hg-Si	La-Sr	Mn-Pb	Pr-V	
Al-In	Be-Mg	Ca-Sc	Cr-La	Er-K	Hf-Mg	La-Ti	Mn-Sr	Pr-Zr	
Al-K	Be-Na	Ca-Sm	Cr-Li	Er-Na	Ho-K	La-V	Mn-Tl	Sc-V	
Al-Na	Be-Se	Ca-Tb	Cr-Mg	Er-Sr	Ho-Mo	La-Zr	Mn-Yb	Sc-Sr	
Al-Pb	Be-Sn	Ca-Ti	Cr-Na	Er-V	Ho-Na	Li-Cs	Mo-Na	Sc-V	
Al-Tl	Be-Sr	Ca-Tm	Cr-Nd	Eu-Li	Ho-Sr	Li-Fe	Na-Nd	Se-Tl	
As-Tl	Be-Zn	Ca-V	Cr-Pb	Eu-Mn	Ho-Ti	Li-K	Na-Ni	Si-Tl	
Au-B	Bi-Co	Ca-Y	Cr-Pm	Eu-Na	Ho-V	Li-Na	Na-Pm	Sm-Sr	
Au-Ir	Bi-Cr	Ca-W	Cr-Pr	Eu-T I	In-Te	Li-Nd	Na-Pr	Sm-Ti	
Au-Mo	Bi-Fe	Ca-Zr	Cr-Sm	Eu-V	In-V	Li-Ni	Na-Sc	Sm-V	
Au-Rh	Bi-Ga	Cd-Cr	Cr-Sn	Eu-Zr	K-La	Li-Pm	Na-Sm	Sn-V	
Au-Rh	Bi-Mn	Cd-Fe	Cr-Sr	Fe-In	K-Li	Li-Pr	Na-Tb	Sn-W	
Au-Se	Bi-Rb	Cd-Ga	Cr-Tm	Fe-K	K-Lu	Li-Rb	Na-Ti	Sn-Zr	
Au-W	Bi-Se	Cd-K	Cr-Y	Fe-Li	K-Mg	Li-Sc	Na-Tm	Sr-Tb	
Au-Ru	Bi-Si	Cd-Na	Cr-Yb	Fe-Mg	K-Mn	Li-Sm	Na-V	Sr-Ti	

**Table 2:** Ternary alloy systems that contain a stable liquid miscibility gap.

Ag-Al-Pb	[6]	Al-Mg-Mn	[6]
Ag-Co-Pd	[6]	Au-Cu-Pb	[6]
Ag-Cu-Fe	[68]	Au-In-Pb	[6]
Ag-Cu-Mn	[68]	Au-In-Pb	[6]
Ag-Cu-Ni	[68]	B-Cu-Fe	[13,69]
Ag-Cu-Pb	[68]	Bi-Ga-Zn	[6]
Ag-Cu-Se	[68]	Co-Cr-Cu	[67,70,71,72]
Ag-Cu-Ti	[68]	Co-Cr-Nb	[67]
Ag-Fe-Mn	[6]	Co-Cu-Fe	[7,8,9,10,11,12,14,15,16,17,73]
Ag-Fe-Ni	[6]	Cr-Cu-Fe	[11,74]
Ag-Nb-Ti	[75]	Cu-Fe-Mo	[76]
Ag-Ni-Sn	[6]	Cu-Fe-Nb	[76]
Al-Bi-Cu	[77]	Cu-Fe -Si	[11,13]
Al-Bi-Sb	[78]	Cu-Fe-Sn	[79]
Al-Bi-Sn	[80,81,82,83]	Cu-Fe-V	[11]
Al-Cu-Sn	[84]	Cu-Ni-Pb	[6]
Al-Ga-In	[6]	Fe-Si-Zn	[6]
Al-Ga-Sn	[6]	Pb-Pd-Sn	[6]

**Table 3:** Multicomponent alloy systems with reported liquid miscibility gaps.

System	Classification	Type of LPS	Ref.
Ag-Al-Co-Cr-Cu-Fe-Ni	HEA	Stable	[107]
Ag-Al-Co-Cr-Cu-Ni	HEA	Stable	[106,107]
Al-Co-Ce-La-Zr	Bulk Metallic Glass	Stable/Metastable	[110]
Al${}_{0.5}$-Co-Cr-Cu-Fe-V	HEA	Stable	[111]
Al-Cr-Cu-Fe-Ni	HEA	Stable	[99]
Al-Cu-La-Ni-Zr	Bulk Metallic Glass	Stable	[112]
B-Cu-Fe-P-Si	Fe-Cu-alloy	Stable	[113]
Co-Cr-Cu-Fe	HEA	Stable	[71,111]
Co-Cr-Cu-Fe-Mn	HEA	Stable	[71]
Co-Cr-Cu-Fe-Mo-Ni	HEA	Stable	[114]
Co-Cr-Cu-Fe-Ni	HEA	Metastable	[115,116,117,118]
Co-Cr-Cu-Fe-Ni-Nb	HEA	Stable	[111]
Co-Cr-Cu-Fe-Ti-V	HEA	Stable	[111]
Co-Cr-Cu-Fe-V	HEA	Stable	[71,111]
Co-Cr-Cu-Mn	HEA	Stable	[71]
Co-Cr-Cu-Mn-V	HEA	Stable	[71]
Co-Cr-Cu-Ni-V	HEA	Stable	[71]
Co-Cr-Cu-V	HEA	Stable	[71]
Cr-Cu-Fe-Mn-V	HEA	Stable	[111]
Cr-Cu-Fe-Mo-Ni	HEA	Stable	[119]
Cr-Cu-Fe -Ni	Cu-alloy	Stable/Metastable	[7]
